# Rosmarinic acid attenuates hepatic steatosis by modulating ER stress and autophagy in oleic acid-induced HepG2 cells

**DOI:** 10.1039/c8ra02849d

**Published:** 2018-07-25

**Authors:** Govindaraj Jayanthy Balachander, Sorimuthupillai Subramanian, Kaliappan Ilango

**Affiliations:** Molecular Biology Division, Interdisciplinary Institute of Indian System of Medicine (IIISM), SRM Institute of Science and Technology Kattankulathur 603203 India ilangok67@gmail.com +91 9444144120; Department of Biochemistry, University of Madras, Guindy Campus Guindy Chennai 600025 India

## Abstract

Non-alcoholic fatty acid disease (NAFLD) has become an emerging entity of liver disorders worldwide. Oxidative stress and deranged autophagy-induced endoplasmic reticulum (ER) stress has recently been recognized as one of the prime factors involved in the pathological mechanism underlying NAFLD and progressive non-alcoholic steato-hepatitis (NASH). Epidemiological and experimental data reveal the potency of dietary polyphenols in averting NAFLD. In this line, to analyse and address the underlying pathogenic mechanisms, in the present study, oleic acid-induced HepG2 cells were treated with rosmarinic acid (RA), a dietary polyphenol with well-established cytoprotective properties. Treatment with rosmarinic acid (20 μg) was found to potently counter the elevated levels of total cholesterol (TC) and triglycerides (TG). Additionally, exposure of oleic acid-induced HepG2 cells to rosmarinic acid showed reduced levels of ROS and increased activity of enzymic and non-enzymic antioxidants. The steatotic HepG2 cells presented a pronounced increase in the expression of key ER stress markers such as p-PERK, p-IRE-1, ATF-6, p-eIF-α and CHOP, which was considerably reduced upon treatment with rosmarinic acid. Moreover, exposure to rosmarinic acid altered the deranged autophagic mechanism in oleic acid-induced HepG2 cells, which was observed *via* the protein expression of Beclin 1, LC31, ATG5 and ATG7. This study demonstrates that rosmarinic acid abrogates NAFLD *via* diminishing ER stress by nullifying oxidative stress and restoring deranged autophagy and can be used as a potent adjunct in the treatment of NAFLD, thus illustrating the valuable application of polyphenols in combating NAFLD.

## Abstract 2

Non-alcoholic fatty acid disease (NAFLD) has become an emerging entity of liver disorders worldwide.

## Graphical abstract

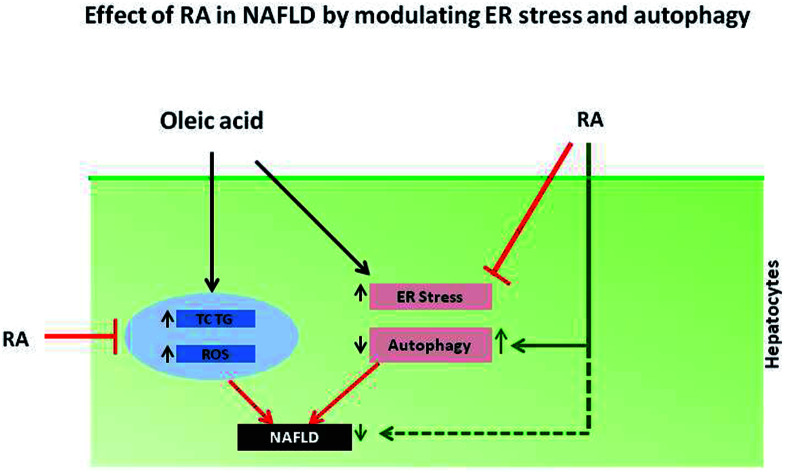

## 1. Introduction

The global shift towards sedentary life style has left a massive imbalance in the energy homeostasis, endorsing a pandemic of metabolic diseases, collectively referred to as metabolic syndrome (MS). Non-alcoholic fatty liver disease (NAFLD), characterised by marked liver pathology, is recognised as the hepatic manifestation of MS and a prominent cause of chronic liver disease in adults and children.^1^ As a condition of increased hepatic fat accumulation, NAFLD occurs as a spectrum of liver injuries ranging from simple steatosis to more severe non-alcoholic steatohepatitis (NASH) with pronounced lobular inflammation and hepatocyte swelling, which might progress to cirrhosis and ultimately hepatocellular carcinoma.^2^ The past decade has seen a drastic increase in NAFLD worldwide, affecting about 90% of obese individuals and 25% of general population, which makes it an emerging epidemic.^3^ Studies also demonstrate that this increase in the occurrence of NAFLD is directly proportional to the inclination towards unhealthy foods rich in sugar and saturated fats, besides genetic predisposition.^4,5^

The progression of NAFLD is currently explicated by “multiple-hit” hypothesis, but the precise mechanism remains to be feebly understood. Recently, ER stress has been implicated in the development of steatosis and progression to NASH. Accumulation of lipids, which is the hallmark of NAFLD, disrupts the ER homeostasis, leading to the activation of intracellular stress pathways and inflammation and culminating in the hepatic cell death. In addition, studies carried out in obese mice show that the derangement of hepatic autophagy also contributes to ER stress, and eventual improvement in ER homeostasis occurred upon restoration of autophagy.^6^

The lack of systemic and validated results poses a hindrance in the development of a better treatment strategy for NAFLD, while the current therapy is more directed towards weight loss and increase in insulin sensitivity through exercise and dietary regimes rather than targeting the disease itself.^7,8^ Over the past few years, medicines from natural sources have earned mounting attention as prospective therapeutic agents to treat NAFLD, accounting for their higher efficacy and lower risk of side effects.^9^

In this line, recent studies have elucidated the protective role of polyphenols in modulating several phenotypic components of NAFLD.^10^ Rosmarinic acid (RA) is an important polyphenol, which has pronounced pharmacological benefits such as antioxidant, antirheumatic and anti-tumour effects.^11^ Evidences also suggest the potent hepato-protective effects of RA, as observed *in vitro*,^12,13^ and also from *in vivo* studies conducted on animals with *tert*-butyl hydroperoxide-, ischemia/reperfusion-, and carbon tetrachloride-induced liver diseases.^14–16^ Further, the study carried out in animal models of extrahepatic cholestasis also ascertained the potency of RA with reference to liver diseases.^17^ Although the beneficial effect of RA against alcohol-induced hepatotoxicity *in vivo* is elucidated,^18^ the mechanistic insight of its action on non-alcoholic hepato-steatosis is not fully explored.

This study was focussed on evaluating the effect of rosmarinic acid on oleic acid-induced NAFLD *in vitro* using HepG2 cells. The suppression of ER stress with augmented autophagy was observed, which could be the speculated mechanism of action of rosmarinic acid in recuperating NAFLD.

## 2. Materials and methods

### 2.1. Chemicals

Rosmarinic acid used in this study was purchased from Sigma-Aldrich (St. Louis, Mo., U.S.A.) and the purity is ≥ 95% (HPLC). Oil red O, oleic acid (OA) and the antibodies for the ER stress and autophagy markers were obtained from Santa Cruz Biotechnology (Santa Cruz, Calif., U.S.A.). Anti-GAPDH antibody was purchased from Sigma-Aldrich.

### 2.2. Cell culture and treatment

HepG2 cells were purchased from NCCS, Pune and grown in Dulbecco's modified Eagle's medium (DMEM) supplemented with 10% fetal bovine serum (FBS), 100 μg mL^−1^ penicillin, 100 μg mL^−1^ streptomycin, and 2 mM l-glutamine. The cells were cultured at 37 °C in a humidified atmosphere of 95% air and 5% CO_2_. The cells were subsequently passaged every four days by trypsinization with 0.25% trypsin–EDTA solution and were treated with 1 mM oleic acid (OA) for induction of steatosis.^19^ RA at a concentration of 20 μM was added to the cells in the presence of OA. Metformin (200 μM) was used as the positive control.

### 2.3. Biochemical analysis

#### 2.3.1. Quantification of steatosis by oil red O staining

The HepG2 cells were cultured in a 96-well culture plate and upon reaching confluence, the cells were washed with PBS and added to a medium containing oleic acid–bovine serum albumin (OA–BSA) complex (4 : 1). Then, the cells were further incubated for 24 h. The cells in the medium containing only BSA were used as the control. The extent of steatosis was quantified by oil Red O (ORO)-based colorimetric assay.^20^

#### 2.3.2. Assay of TC and TG

After 24 h of treatment with experimental drugs, the cells were washed thrice with PBS and lysed with 1% triton X-100 in PBS. The cell lysate were centrifuged at 10000*g* for 2 min, and the supernatant was collected and assayed for TG using a commercially available enzymatic kit (Reckon Diagnostics, Baroda, India). The results were expressed as percentage TG. These values were normalized to total protein in the extract, measured by the Bradford reagent method (Bio-Rad).

#### 2.3.3. Assay of AST and ALT

The ALT and AST levels in the cell supernatants were investigated using commercial kits following the manufacturer's instructions (BIORAD).

#### 2.3.4. Assay of albumin, urea and alanine amino transferase (ALAT)

Cell supernatants were collected for analysis of albumin, urea nitrogen and alanine transferase (ALAT) and analyzed using commercial assay kits obtained from Diasys Diagnostics Systems (Holzheim, Germany) following the manufacturer's instructions.

#### 2.3.5. Determination of intracellular ROS formation

Intracellular ROS level was detected using the fluorescence probe DCFH-DA. HepG2 cells were seeded in a 24-well plate (5 × 10^4^ cells per well) and incubated with rosmarinic acid for 24 h. Subsequently, 150 mM ethanol was added to the cells, and they were then incubated with 30 μM DCFH-DA for an additional 30 min at 37 °C. The fluorescence intensity of the cells was measured using a fluorescence microplate reader at an excitation wavelength of 485 nm and an emission wavelength of 530 nm.

#### 2.3.6. Lipid peroxidation

The degree of lipid peroxidation was estimated by the levels of malondialdehyde measured using the thiobarbituric acid reactive substances (TBARS) assay at 535 nm.^21^

#### 2.3.7. Assay of enzymic and non-enzymic antioxidants

The HepG2 cells were collected and homogenized in 50 mM phosphate buffer containing 1.15% KCl. The homogenized suspension was centrifuged at 13 000*g* for 30 min at 4 °C, and the obtained supernatant was used for the measurement. The activities of CAT, GST, GPx, and SOD and the level of GSH and GSSG were determined according to the method described by You *et al.* in 2010.^22^

### 2.4. Protein extraction and western blotting

Total protein lysates from cells were extracted in RIPA lysis buffer. The protein concentration was determined using a Bradford protein assay kit (Bio-Rad, USA). Equal amounts of protein (50 μg) were separated using 10% SDS–polyacrylamide gel electrophoresis and transferred onto PVDF membranes. The membranes were blocked at room temperature (RT) for 1 h in PBST (PBS and 0.05% Tween 20) containing 5% BSA and probed with 1 : 1000 primary antibodies. Then, the membranes were blocked in PBS and 5% non-fat dried milk containing 0.05% Tween 20 and subjected to incubation with a primary antibody (in 5% BSA - TBST, overnight at 4 °C) and a HRP-conjugated secondary antibody (in 5% BSA, TBS, 2 h at RT). Finally, the protein–antibody complexes were visualised using horseradish peroxidase conjugates with DAB as the substrate. The protein levels of β-actin were used as an internal control for equal loading. The gel image was scanned using an imaging system (Bio-Rad) and quantified.

### 2.5. Reverse transcriptase PCR

Total RNA was obtained from the cells using the TRIzol reagent (TaKaRa, Tokyo, Japan) following the manufacturer's instruction. Total RNA was resuspended in RNase-free solution, quantified by spectrophotometry at an absorbance of 260 nm, and qualitatively assessed by gel electrophoresis. The purified RNA was reverse transcribed to produce cDNA by gel electrophoresis using a Reverse Transcription kit (TaKaRa, Tokyo, Japan). Semi-quantitative RTPCR was performed using Emerald Amp PCR Master Mix (TaKaRa, Tokyo, Japan) following the manufacturer's instructions. The specific primers used for PCR are listed in Table 1. Each sample was run in triplicate under specific conditions, and PCRs without the addition of the template strands were used as blank controls. The relative quantitative values of each sample were normalised with a loading control.

**Table 1:** Primer sequences

P-PERK	Forward	ATCCCCCATGGAACGACCTG
	Reverse	ACCCGCCAGGGACAAAAATG
IRE1α	Forward	GAAGACGTCATTGCACGTGAATT
	Reverse	AGGTCCTGAATTTACGCAGGT
ATF6	Forward	GCTTCCAGCAGCACCCAAGAC
	Reverse	CGTCTGGCCTTTAGTGGGTGCA
CHOP	Forward	CAGAGGTCACAAGCACCT
	Reverse	TCCCTGGTCAGGCGCTC
β-actin	Forward	GGACTTCGAGCAGGAGATGG
	Reverse	GCACCGTGTTGGCGTAGAGG

### 2.6. Statistical analysis

The results were analyzed using one-way analysis of variance (ANOVA) and Student's *T*-test to determine the level of significance. *P* < 0.05 was considered to be significant. The results were expressed as mean ± SD. The statistical analysis was carried out using the SPSS16 software.

## 3. Results

### 3.1. Reduction of levels of TC and TG in HepG2 cells by RA

**Fig. 1:**
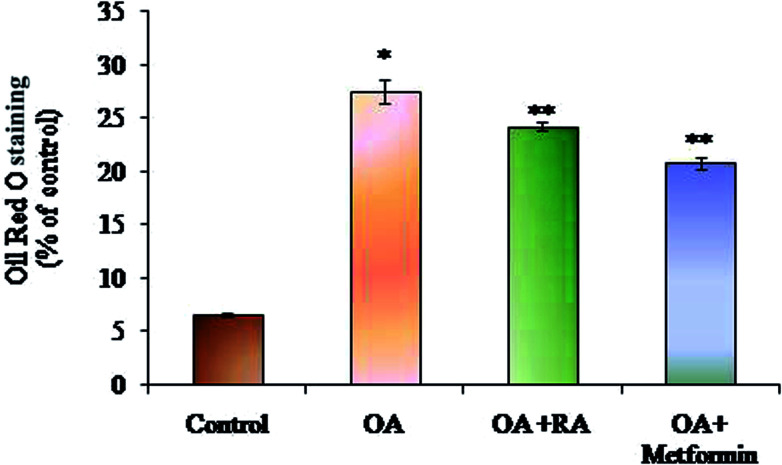
Effect of rosmarinic acid on the lipid accumulation in HepG2 cells. Values are expressed as mean ± SD. The values are statistically significant at *P* < 0.05. *Compared with the control cells; **compared with the OA-induced cells.

**Fig. 2:**
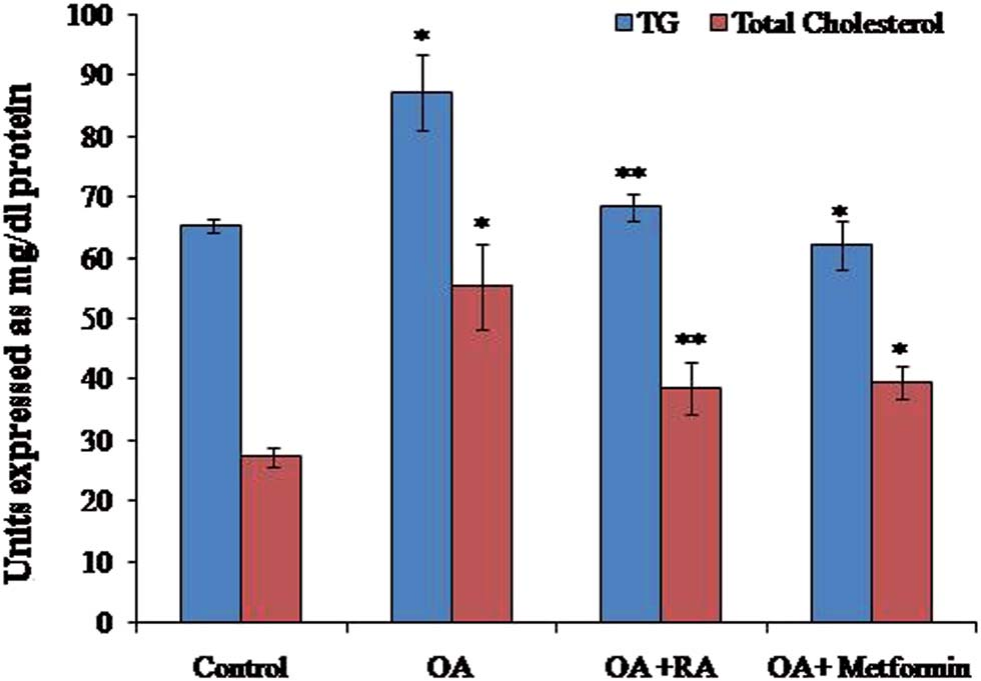
Effect of rosmarinic acid on the levels of TC and TG in oleic acid-treated HepG2 cells. TC and TG are expressed in mg dl^−1^. Values are expressed as mean ± SD. The values are statistically significant at *P* < 0.05. *Compared with the control cells; **compared with the OA-induced cells.

Exposure of oleic acid-treated cells to RA substantially recovered the oil Red O content (Fig. 1), indicating its anti-steatotic effect. Further, treatment with rosmarinic acid also decreased the elevated TC and TG contents in steatotic HepG2 cells, which was similar to that observed in steatotic cells treated with metformin (Fig. 2).

### 3.2. Effect of RA on biochemical markers in OA-induced HepG2 cells

Addition of oleic acid to HepG2 cells showed a considerable decrease in urea and albumin secretion, whereas an increase in ALAT activity and AST and ALT levels was observed when compared to those observed for control cells. However, addition of RA decreased the ALAT activity and AST and ALT levels (Table 2), while increasing the amount of urea and albumin secreted into the cell supernatant when compared to those of OA-treated cells.

**Table 2:** Effect of rosmarinic acid on the various biochemical parameters in HepG2 cells

Parameters	Control	OA	OA + RA	OA + metformin
AST (IU L^−1^)	60.56 ± 1.21	125.23 ± 2.62*	89.78 ± 2.76**	80.19 ± 2.32**
ALT (IU L^−1^)	23.22 ± 0.95	28.34 ± 1.64*	25.52 ± 1.19**	24.86 ± 0.96**
Urea (μM)	0.92 ± 0.04	0.63 ± 0.03*	0.79 ± 0.05**	0.81 ± 0.05**
Albumin (mg mL^−1^)	0.94 ± 0.04	0.51 ± 0.02*	0.98 ± 0.04**	0.92 ± 0.05**
ALAT (units per mL)	4.10 ± 0.18	10.74 ± 0.55*	8.22 ± 0.36**	9.56 ± 0.39**

### 3.3. Effect of RA on ROS levels in HepG2 cells

**Fig. 3:**
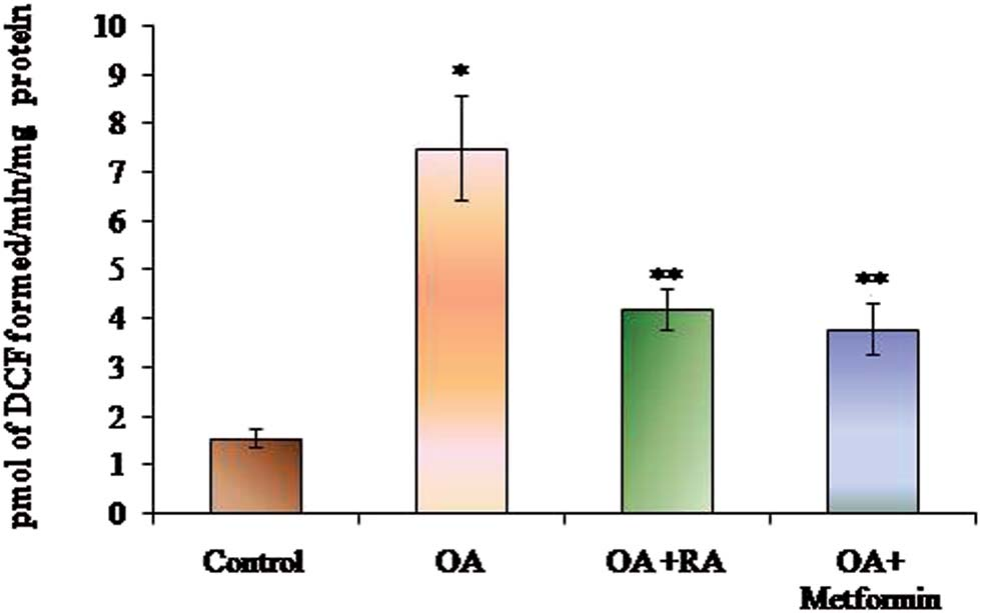
Effect of rosmarinic acid on the level of ROS during oleic acid-induced steatosis in HepG2 cells. Units are expressed in pmol DCF formed per min per mg protein. Values are expressed as mean ± SD. The values are statistically significant at *P* < 0.05. *Compared with the control cells; **compared with the OA-induced cells.

The efficacy of RA on the levels of ROS generated is depicted in Fig. 3. The HepG2 cells cultured in media containing oleic acid had an elevated ROS generation when compared to the control cells. Moreover, treatment of the steatotic HepG2 cells with RA as well as metformin suppressed the ROS levels, indicating the antioxidant capacity of RA.

### 3.4. Improvement of the antioxidant status in HepG2 cells by RA

Table 3 and 4 illustrate that RA effectively augments the activities of both enzymic (SOD, CAT, and GPx) and non-enzymic antioxidants (GSH and GSSG) in steatotic HepG2 cells. This is consistent with the reduced ROS production observed in the oleic acid-induced HepG2 cells treated with RA.

**Table 3:** Effect of rosmarinic acid on the enzymic antioxidant status in HepG2 cells in the steatotic state

Groups	SOD^a^	CAT^b^	GPx^c^
Control	35.68 ± 1.28	0.75 ± 0.03	21.58 ± 1.16
OA	14.97 ± 0.86*	0.38 ± 0.026*	8.97 ± 0.59*
OA + RA	24.64 ± 1.23**	0.64 ± 0.041**	19.68 ± 1.55**
OA + metformin	26.62 ± 1.10*	0.69 ± 0.032*	18.32 ± 1.20*

**Table 4:** Effect of rosmarinic acid on the non-enzymic antioxidant status in steatotic HepG2 cells

Groups	GSH (nmoles mg^−1^ protein)	GSSG (nmoles GSH equiv. mg^−1^ protein)
Control	69.75 ± 2.30	5.45 ± 1.7
OA	25.25 ± 1.48*	32.34 ± 1.8*
OA + RA	38.76 ± 1.78**	14.67 ± 4.8**
OA + metformin	47.14 ± 1.55*	17.58 ± 3.5*

### 3.5. Effect of rosmarinic acid on the ER stress adaptation in HepG2 cells

**Fig. 4:**
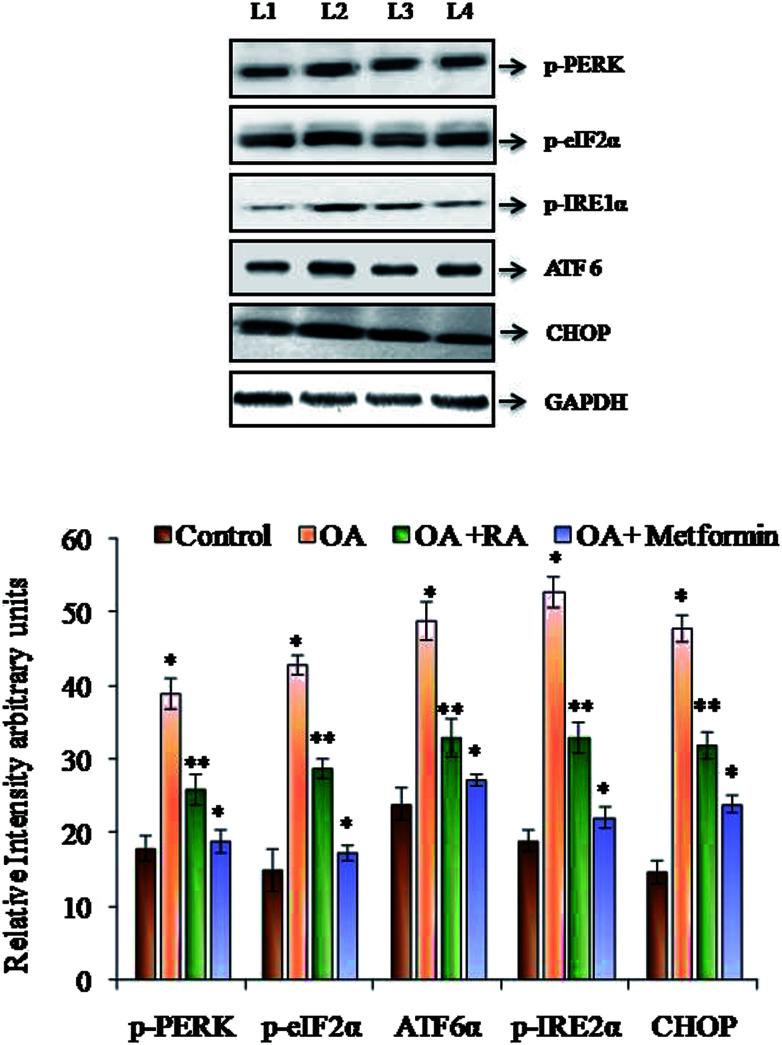
Representative immunoblots of the various ER stressors in HepG2 cells exposed to oleic acid. L1: control; L2: oleic acid; L3: oleic acid + RA; and L4: oleic acid + metformin. Values are expressed as mean ± SD. The values are statistically significant at *P* < 0.05. *Compared with the control cells; **compared with the OA-induced cells.

**Fig. 5:**
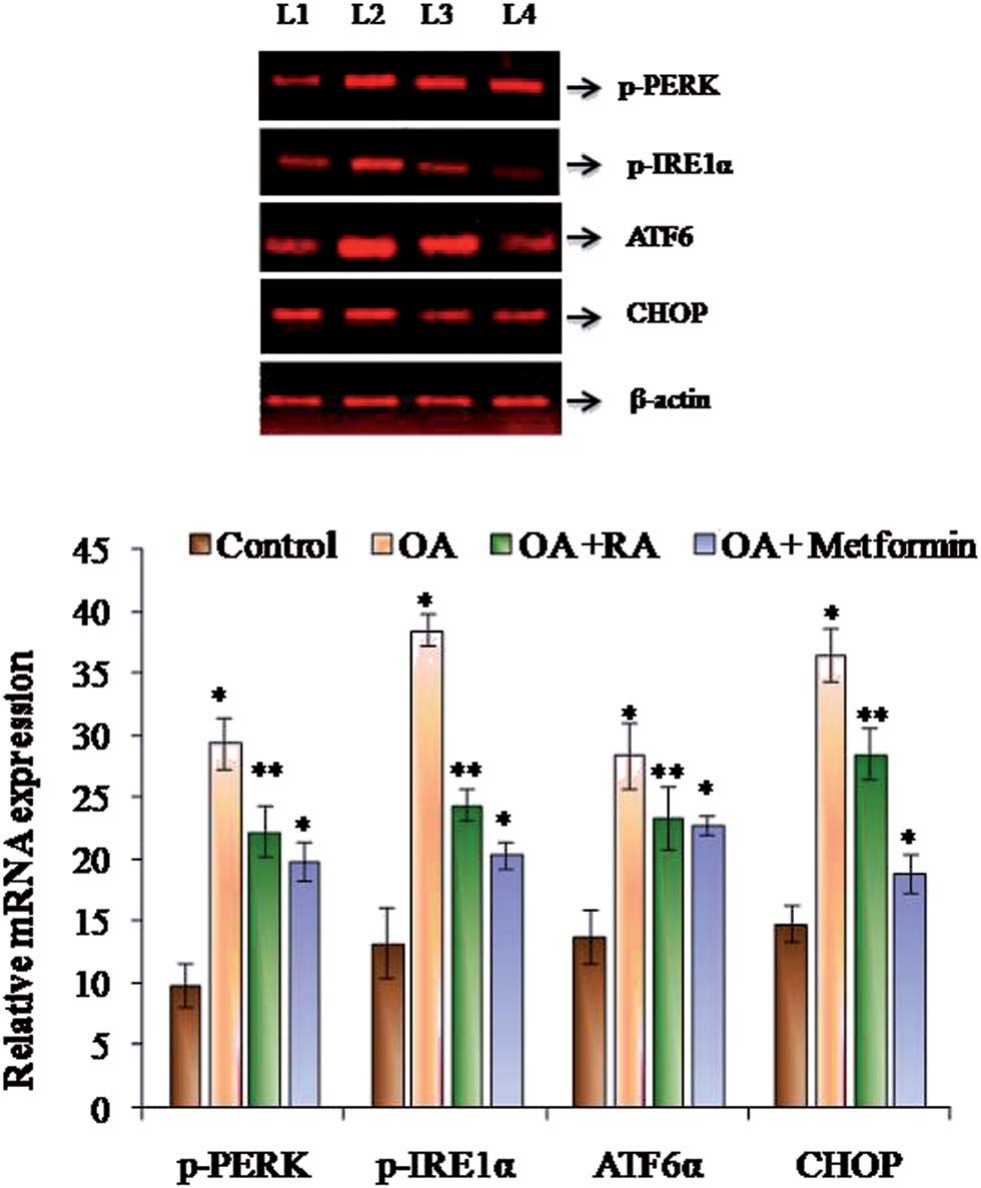
Representative mRNA expressions of p-PERK, p-IRE1α, ATF6 and CHOP in oleic acid-treated HepG2 cells. L1: control; L2: oleic acid; L3: oleic acid + RA; and L4: oleic acid + metformin. Values are expressed as mean ± SD. The values are statistically significant at *P* < 0.05. *Compared with the control cells; **compared with the OA-induced cells.

It was observed (Fig. 4) that RA treatment of the HepG2 cells cultured in oleic acid medium significantly diminished the protein expression of p-PERK, p-IRE1α and ATF6, the three main ER stressors, along with that of p-eIFα and CHOP. The gene expression analysis (Fig. 5) in oleic acid-induced hepatocytes also showed the same observation as that of the abovementioned analysis, thus validating the effect of RA as a potent douser of ER stress, which is the main pathogenic event of NAFLD.

### 3.6. Effect of rosmarinic acid on the autophagic dysregulation in HepG2 cells

**Fig. 6:**
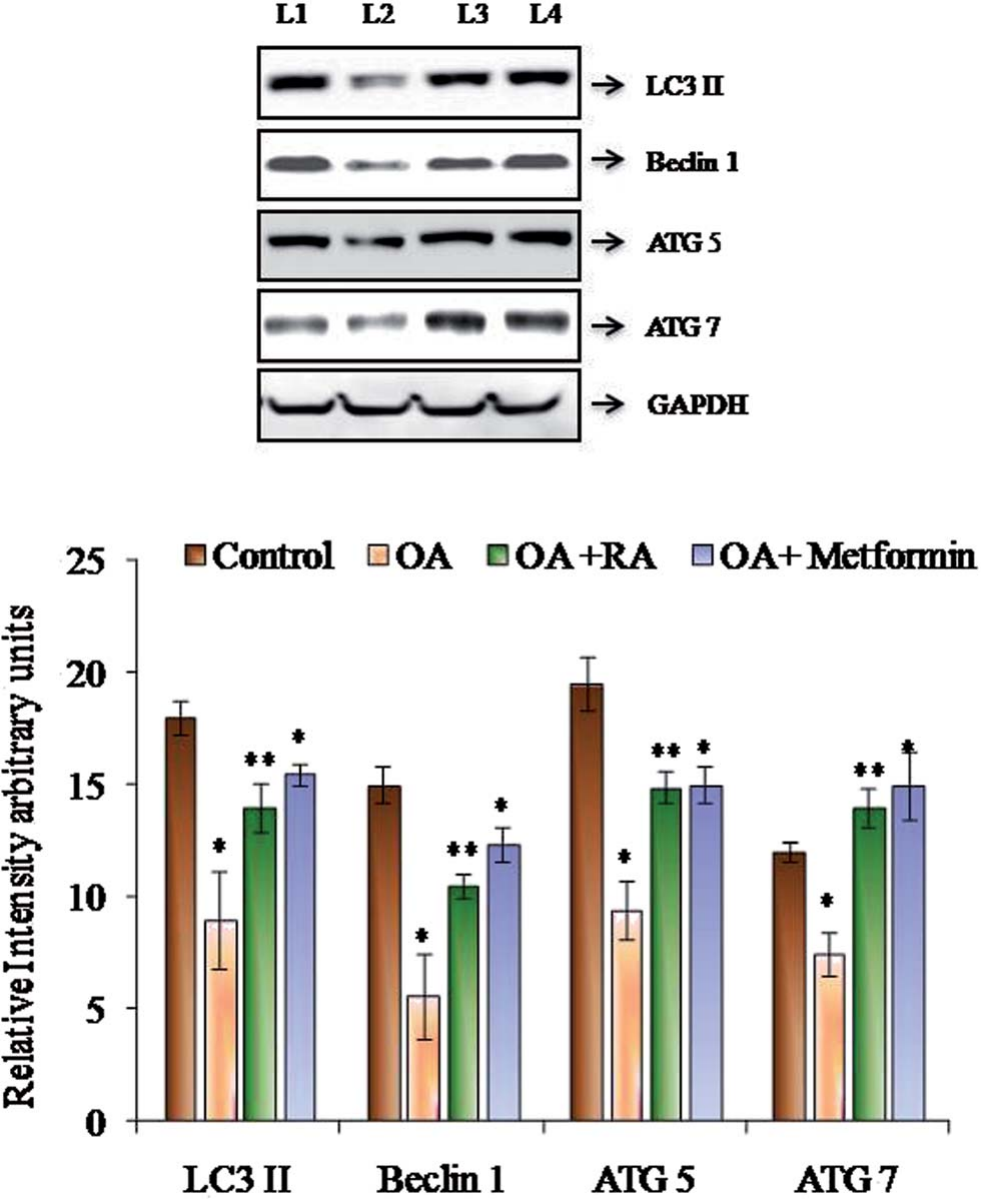
Representative immunoblots of the autophagic markers in HepG2 cells exposed to oleic acid. L1: control; L2: oleic acid; L3: oleic acid + RA; and L4: oleic acid + metformin. Values are expressed as mean ± SD. The values are statistically significant at *P* < 0.05. *Compared with the control cells; **compared with the OA-induced cells.

Fig. 6 shows the western blot analysis of the autophagic markers in the control and oleic acid-induced cells treated with RA. Pronounced suppression of the major autophagic proteins such as LC3II, Beclin-1, Atg-5 and Atg-7 was observed in the HepG2 cells treated with oleic acid. Moreover, treatment with rosmarinic acid as well as metformin radically restored the deranged expression of the abovementioned markers, which indicates the competence of RA in battling NAFLD.

## 4. Discussion

Prior documented reports have predicted that NAFLD turns out to be the epidemic of the decade, as it is becoming the leading cause of chronic liver disease worldwide and NASH is projected to be the most common indication of the need for liver transplantation. Apart from the association of NAFLD with chronic liver disease, kidney disease and cardiovascular disease,^23–25^ there is increasing evidence that it is linked to other persistent conditions such as sleep apnea, colorectal cancer, osteoporosis, psoriasis and various endocrinopathies.^26^ This demands an inevitable need to explore effective approaches for NAFLD treatment.

The development of effective therapeutic strategies for NAFLD treatment is of urgent need as there are no approved pharmacological therapies and the main clinical advice as an initial step is lifestyle modification.^27^ The numerous side effects and impediments of the currently available drugs for NAFLD such as glitazones lead to the inclination towards alternative treatment approaches.^28–31^ Extensive studies have explored the role of specific nutrients and phytochemicals against NAFLD,^32,33^ and polyphenols are speculated to be an important and compelling nutraceutical in preventing NAFLD-associated pathologies.^34^ Among the polyphenolic substances, RA has attracted considerable interest due to its important therapeutic properties and health benefits. It is an ester of caffeic acid and 3,4-dihydroxyphenyllactic acid. The wide array of pharmacological properties of RA are thought to be based on enhancement of superoxide and hydroxyl scavenging activities, inhibition of both low-density lipoprotein and oil oxidation, and inhibition of haemolysis and hyaluronidase and β-hexosaminidase activities.^35^ It is contemplated that polyphenols present the hepatoprotective effect by increasing fatty acid oxidation and modulation of insulin resistance, oxidative stress and inflammation.^36^ Based on the abovementioned findings, we investigated the role of RA in ameliorating NAFLD in context with its well-established *in vivo* and *in vitro* hepatoprotective effects in improving insulin sensitivity and dampening oxidative stress-mediated injuries.^37–39^

In the process of identifying potent treatment options, exploration of molecular targets to combat NAFLD is also emerging along with other therapies such as dietary intervention.^40,41^ Moreover, proper understanding of the various pathophysiological processes underlying NAFLD becomes unavoidable for its anticipated strategic remedial measures. In this line, studies postulate ER stress as an important pathogenic mechanism in the manifestation of NAFLD^42^ and currently, the role of ER stress in NAFLD is extensively investigated. ER is one of the largest cellular organelles, and it functions to properly arrange the intracellular organelles and cell surface proteins.^43^ Any perturbations in ER homeostasis leads to the accumulation of unfolded proteins and protein aggregates in the ER lumen, thus aggravating ER stress.^44^ Cells encounter the early stressed state by activating the unfolded protein response (UPR), which scrutinizes and counters the accumulation of inappropriately folded proteins in the ER lumen.^45^ However, long-lasting ER stress proves to have detrimental effects on metabolic functions, thus leading to obesity, type 2 diabetes, NAFLD and NASH.^46^ This finding has been corroborated by several studies carried out in various *in vivo* models, in which persistent UPR was ascribed to hepatic steatosis.^47–50^ Furthermore, disturbed autophagy is also considered as a key contributor of ER stress, and earlier reports have established that a putative link between ER stress and autophagy contribute to the pathogenic transformation of NAFLD.^51^

Several presented studies hint on the effect of polyphenols in attenuating ER stress in hepatic, adipose and skeletal muscle tissues, and most of their results display promising effects.^52^ In this conjecture, the present study aimed to analyse the potency of RA in assuaging ER stress in oleic acid-induced hepatic steatosis. Initially, it was found that RA addition to oleic acid-induced cells restored the levels of urea and albumin, signifying its cell proliferative capacity. Similarly, the decrease in ALAT activity in steatotic cells by RA corroborates its hepatoprotective state during non-alcoholic steatosis.^53^ Further, steatotic cells treated with RA exhibited decrease in the levels of total cholesterol and triglycerides, which was corroborated by the quantification of oil red O staining of the cells.

ROS are recognized intermediaries of intracellular signalling cascades, whose excessive production leads to oxidative stress, loss of cell function and apoptosis or necrosis. Steatotic HepG2 cells treated with RA hindered ROS production, as revealed in the present study. This observation is in concurrence with the earlier published reports of the effect of RA on oxidative stress in type 2 diabetes.^54^ RA is well known for its pronounced antioxidant properties, which is attributed to its four phenolic groups and two catechol moieties.^55^ In the present study, the exposure of the oleic acid-treated cells to RA showed increased levels of both enzymic and non-enzymic antioxidants, underlying its antioxidant efficacy, the result of which was similar to that observed in cells treated with metformin.

In addition, studies by Quan *et al.* revealed that ROS generation is an early event that triggers ER stress.^56^ Data obtained from various reports suggest that oxidative stress and ROS generation are not only a consequence of ER stress induction, but also a fundamental component of ER stress.^57,58^ Thus, in the present study, the increase in production of ROS and lipid peroxidation in the oleic acid-treated HepG2 cells signifies the induction of ER stress, thus appending to the pathogenicity of NAFLD. ER stress in NAFLD and steatosis generally plays a vicious role directly or indirectly *via* the induction of *de novo* lipogenesis and perturbation of mitochondrial biogenesis.^59^ Oleic acid induction to HepG2 cells was found to increase the protein and gene expression of p-PERK, p-IRE-1, ATF 6 and p-eIF2α, a downstream target of PERK and CHOP. Moreover, treatment of steatotic HepG2 cells with RA was found to downregulate the expression of the ER stressors, which was similar to that observed in the study performed with resveratrol and berberine.^60,61^ IRE1α and PERK phosphorylation functions as direct markers of UPR activation and their induction indirectly promote *de novo* lipogenesis. This was substantiated by the study where silencing of PERK inhibits the expression of prolonged lipogenic enzymes such as FAS and ATP citrate lyase.^62^ Similarly, hepatocyte-specific IRE1α knockout mice developed severe hepatic steatosis following treatment with an ER stress inducer *via* repressing expression of key metabolic transcriptional regulators and enzymes associated with triglyceride biosynthesis.^63^ In addition, the ATF6 pathway also plays an important role in stress-related lipid deposition, which was demonstrated in an *in vivo* experiment, in which ATF-6α-knockout mice developed hepatic steatosis in response to an ER stress inducer as a consequence of reduced fatty acid β-oxidation and decreased VLDL production.^64^ It is also reported that a surge in ROS can effectively induce apoptosis, apparently *via* the activation of ER-stress-mediated apoptosis. CHOP is a UPR-induced stressor that intercedes ER-stress-mediated apoptosis,^65^ and antioxidant treatment in crisimartin induced UPR activation in gall bladder tumours abolished the expression of CHOP induced by crisimartin.^66^ Thus, collective results depict that UPR induction in the milieu of fatty liver apparently intensifies ER stress in a counter-protective manner and promotes hepatic steatosis with varying substantial contribution of the different UPR branches.^67^

In addition to the role of ROS in inducing ER stress, *in vivo* studies performed by Yang *et al.*^68^ showed that deranged autophagy also contributes to ER stress, and the restoration of autophagy improved ER homeostasis. Recent data have also suggested that hepatocyte lipid accumulation is associated with diminished autophagic function, which indicates the role of autophagy in regulation of lipid homeostasis in hepatocytes.^69^ In the present study, the protein expression of the LC3II, Beclin 1, ATG5 and ATG7 was decreased by treatment of HepG2 cells with oleic acid. LC3II is the characterized component of autophagosome and its decrease indicates reduced autophagosome turn over, which might lead to the accumulation of lipid droplets in the hepatocytes.^51^ Similarly, Beclin1 is also the key gene in the process of autophagy, which represents the amount of autophagy activity. Moreover, studies show that the increase in calpain-2 levels during obesity leads to degradation of Atg-7, resulting in defective autophagy; also, knockdown of Atg-5 results in an increase in the intracellular triglyceride content, thus alleviating steatotic condition.^66,67^ In the present study, RA was found to restore the expression of the abovementioned genes, indicating its efficacy in regulating autophagy, the effect which was similar to that observed in a study performed with resveratrol.^70^

In conclusion, in the pursuit of developing effectual therapy for NAFLD using polyphenols, RA poses to be a potent supplement, possibly through abrogating oxidative stress-induced ER stress and deranged autophagy. Since the efficacy of RA in alleviating lipogenesis *via* the AMPK pathway during HFD-STZ-induced type 2 diabetes was established in our previous studies,^71^ we here endorse that ER stress and impaired autophagy serve to be potent molecular targets for RA in alleviating NAFLD.

## Conflicts of interest

The authors declare no conflict of interest.

## Supplementary Material
